# Modification of Montmorillonite with Polyethylene Oxide and Its Use as Support for Pd^0^ Nanoparticle Catalysts

**DOI:** 10.3390/polym11050755

**Published:** 2019-04-29

**Authors:** Guiqing Shu, Jing Zhao, Xiu Zheng, Mengdie Xu, Qi Liu, Minfeng Zeng

**Affiliations:** Zhejiang Key Laboratory of Alternative Technologies for Fine Chemicals Process, College of Chemistry and Chemical Engineering, Shaoxing University, Shaoxing 312000, China; guiqingshu@163.com (G.S.); jingjingzhao218@sina.com (J.Z.); xiuzheng2019@163.com (X.Z.); joanne7280@163.com (M.X.); lq920819@163.com (Q.L.)

**Keywords:** polyethylene oxide, montmorillonite clays, Pd catalysis, catalytic composite, positron annihilation

## Abstract

In this study, montmorillonite (MMT) was modified by intercalating polyethylene oxide (PEO) macromolecules between the interlayer spaces in an MMT-water suspension system. X-ray diffraction results revealed that the galleries of MMT were expanded significantly after intercalation of different loading of PEO. MMT/PEO 80/20 composite was chosen as the support platform for immobilization of Pd species in preparing novel heterogeneous catalysts. After immobilization of Pd species, the interlayer spacing of MMT/PEO (80/20) (1.52 nm) was further increased to 1.72 nm (Pd^2+^@MMT/PEO) and 1.73 nm (Pd^0^@MMT/PEO), confirming the well-immobilization of the Pd species in the interlayer spaces of PEO-modified MMT. High-resolution transmission electron microscopy (HR-TEM) observation results confirmed that Pd nanoparticles were confined inside the interlayer space of MMT and/or dispersed well on the outer surface of MMT. The conversion of Pd^2+^ to Pd^0^ species was evidenced by binding energy characterization with X-ray photo electron spectroscopy (XPS). The microstructure variation caused by the Pd immobilization was sensitively detected by positron annihilation lifetime spectroscopy (PALS) studies. The prepared Pd^0^@MMT/PEO (0.2/80/20) catalytic composite exhibits good thermal stability up to around 200 °C, and it showed high activities for Heck reactions between aryl iodides and butyl acrylates and could be recycled for five times. The correlations between the microstructure and properties of the Pd@MMT/PEO catalytic composites were discussed.

## 1. Introduction

The modification of montmorillonite (MMT) with organic polymers has attracted more and more interests for their excellent structural and/or functional performances [1,2,3]. Many water-soluble polymers, such as polyethylene glycol (PEG) [4], polyethylene oxide (PEO) [5], polyvinyl alcohol (PVA) [6], polyvinyl pyrrolidone (PVP) [7], etc., can be easily intercalated in MMT with the water solution intercalation method. In the MMT/polymer nanocomposites, an obvious increase in the interlayer spacing of MMT is frequently observed, indicating the intercalated polymer chains are well-confined in the narrow galleries between the MMT layers. Meanwhile, the lipophilicity of MMT will be significantly improved after the organic polymer chains intercalated. The prepared MMT/polymer hybrid composites are nowadays frequently applied in many fields, such as preparation of advanced nano-composites, drug delivery, and water treatment, etc. [8,9,10]. 

Recently, many researchers shift the attentions towards heterogeneous catalysis topics with use of MMT-based composites [11,12,13,14]. In particular, MMT/polymer composites have been recognized as good supporting materials for immobilization of transition metal catalysts applied in organic reactions [15,16,17,18,19,20,21]. With synergetic processes of ion-exchange with interlayer cations of MMT and complexation with polar polymers, the transition metal cations, like Pd^2+^ will be easily immobilized on MMT/polymer supports. It has been evidenced that polymers (such as PVA, PVP, chitosan (CS), etc.) modified MMT supported Pd catalytic materials has good comprehensive catalytic performances as applied in Heck and/or Sonogashira coupling reactions. Pd species can be tightly encaged in the narrow galleries between the MMT layers. For same Heck reaction, it is reported [15,17] that Pd@MMT/CS can reuse more times (30 times) than Pd@MMT/PVP catalysts (10 times). It was due to the weaker encaging of Pd species for bigger interlayer space formed in the case of Pd@MMT/PVP and lower chelation ability of PVP than that of CS. This revealed that the catalytic performances can be tailored with different kinds of polymers for the different intercalation structure formed and chelation ability with transition metals. 

Polyethylene oxide is another well-known modifier for MMT and the prepared MMT/PEO hybrid composites show potential applications in many fields [22,23]. It was suggested that PEO chains could form single or double-ordered layer arrangements in the interlayer space of MMT. The ending groups of PEO, i.e., –OH groups, have effective chelation capability with transition metal species. Also, PEO is known as an effective stabilizer for transition metal nanoparticles in reducing of their agglomeration to form big-sized particles [24,25]. PEO itself has been used as a polymer support for Pd catalysts in Heck reactions [26,27,28]. For example, PEO-supported recyclable NC palladacycle catalysts were developed by Karami and co-workers [28] by blending of dimeric NC palladacycle with PEO melts at 85 °C. However, the size of the prepared Pd nanoparticles is too large (bigger than 50 nm) and it just showed moderate catalysis efficiency (up to 80% yield) for Heck reactions. Therefore, further modification of PEO-supported Pd catalysts is needed. It is expected that PEO-modified MMT should be another good candidate of support for immobilization of transition metals (such as Pd) in heterogeneous catalytic materials preparation. 

In this study, PEO-modified MMT supports with different content and Pd-supported on MMT/PEO catalytic composites have been prepared. The microstructure of the PEO chains and Pd species confined in the interlayer space of MMT was characterized by several methods, including X-ray diffraction (XRD), high resolution transmission electron microscopy (HR-TEM), X-ray photo electron spectroscopy (XPS), thermal analysis, differential scanning calorimetry (DSC), and inductively coupled plasma-atomic emission spectroscopy (ICP). The sub-nano level microstructure of Pd@MMT/PEO catalytic composites was further studied by positron annihilation lifetime spectroscopy (PALS). The correlations between the microstructure and catalytic performances of Pd@MMT/PEO catalytic composite in Heck reactions were discussed.

## 2. Materials and Methods 

### 2.1. Materials

Na^+^−MMT (G-105 type) of was purchased from Nanocor Co., Arlington Hts, IL, USA and its cationic exchange capacity was 145 mmol/100 g. PEO (*M*_n_ = 2 × 10^4^ g/mol) was purchased from Sinopharm Chemical Reagent Co., Ltd. Shanghai, China. PdCl_2_ (analytical grade) was purchased from Zhejiang Metallurgical Research Institute Co., Ltd. Hangzhou, China. The aromatic halides and acrylate substrates used in Heck reactions were purchased from Energy Chemical, Sun Chemical Technology (Shanghai) Co., Ltd. Shanghai, China. Other used reagents and solvents with no further treatments were purchased from Sinopharm Chemical Reagent Co., Ltd. Shanghai, China.

### 2.2. Sample Preparation 

A total of 100 mL of 2 wt % of MMT suspension solution was prepared under magnetic stirring. Specific amounts of PEO were dispersed in 100 mL of deionized water to form homogeneous solution. The PEO solution and the MMT suspension solution were mixed and kept magnetically stirred in water bath at 55 ° C for 12 h. The mass ratios of MMT/PEO were set as 100/0, 90/10, 80/20, 70/30, 60/40, and 50/50. 0.3 g of PdCl_2_ was dissolved in 100 mL deionized water with the presence of excessive amounts of NaCl (about 2 g). Then, 2 mL of the fresh Pd^2+^ solution was drop-wisely added into the above MMT/PEO (80/20) mixture and kept magnetically stirring for another 6 h. The Pd^2+^@MMT/PEO composite was separated by centrifugation and washed with deionized water until neutral (pH = 7). Afterwards, it was dried in an oven at 60 °C. The resultant Pd^2+^@MMT/PEO composite was reduced to Pd^0^@MMT/PEO with ethylene glycol at 80 °C. According to the ICP-AES determination results, the Pd content within the Pd@MMT/PEO catalytic composite was about 0.2 wt %.

### 2.3. Characterizations

The characterization methods were similar to those in our recent works [19,20,21]. The XRD analysis of MMT/PEO and Pd@MMT/PEO samples were performed with a PANalytical Empyrean X-ray diffraction system (conditions: 2*θ* from 3° to 70°, scanning rate of 2°/min). The TGA and DSC curves of MMT/PEO and Pd@MMT/PEO samples were recorded with a Mettler Toledo TGA/DSC 2 STAR system (Zurich, Switzerland) (conditions: air atmosphere, from 30 to 700 °C, scanning rate of 20 °C/min). The XPS analysis of Pd@MMT/PEO sample was performed with a Thermo Fisher Scientific ESCALAB 250Xi X-ray photoelectron spectrometer. The Pd@MMT/PEO samples were embedded in epoxy resin and then microtomed for HRTEM observation, which was performed with a JEM-2100F HR-TEM (JEOL Ltd. Tokyo, Japan). The ICP determination of Pd@MMT/PEO samples were performed with a Leemann ICP-AES Prodigy XP inductively coupled plasma atomic emission spectrometry. The positron annihilation lifetime spectroscopy (PALS) analysis of MMT/PEO and Pd@MMT/PEO samples was performed with an EG & G ORTEC fast-slow system (conditions: time resolution of 198 ps). Before PALS measuments, the MMT/PEO and Pd@MMT/PEO samples were pressed into disks (diameter×thickness: 1 cm × 2 mm) using a 769YP-15A powder tableting machine (Shanghai Xinnuo Instrument Ltd., Shanghai, China). During lifetime spectra measurements, the positron source (^22^ NaCl, 16 μCi, deposited between two Kapton foils) was sandwiched between two pre-pressed Pd@MMT/PEO samples disks. The analysis of the positron annihilation spectra was performed with LT-9 (Lifetime-9) and MELT-4 (Maximum Entropy for Lifetime Analysis-4) programs.

### 2.4. Catalytic Test

In a 50 mL of round bottom flask reactor, a mixture of aromatic halide substrates (1 mmol), acrylates substrates (2 mmol), Pd^0^@MMT/PEO catalytic composite (3 μmol of Pd), CH_3_COOK base (3 mmol), and solvent (5 mL DMSO + 0.2 mL ethylene glycol) was magnetically stirred at 110 °C (oil bath heating) for 5 h. The coupling reaction progress was detected with both layer chromatography (TLC) method and gas chromatography-mass spectrometry (GC/MS) analysis (Agilent 6890N/5975 MSD GC/MS, Palo Alto, CA, USA). The coupling reaction yield is according to the GC/MS quantitative analysis results of the reaction mixture. All the coupling products’ chemical structure was confirmed by the analysis results of both H^1^ NMR and GC/MS, which was consistent with our recent works [21,29]. The recycling experiments of the Pd@MMT/PEO were performed as follows: firstly, filtration out the Pd@MMT/PEO from the reaction system; secondly, repeatedly washing of the filtrated Pd@MMT/PEO with ethanol for 3–5 times and drying; finally, putting the recycled Pd@MMT/PEO into the reaction mixture for use in next reaction run. 

## 3. Results and Discussions

**Figure 1:**
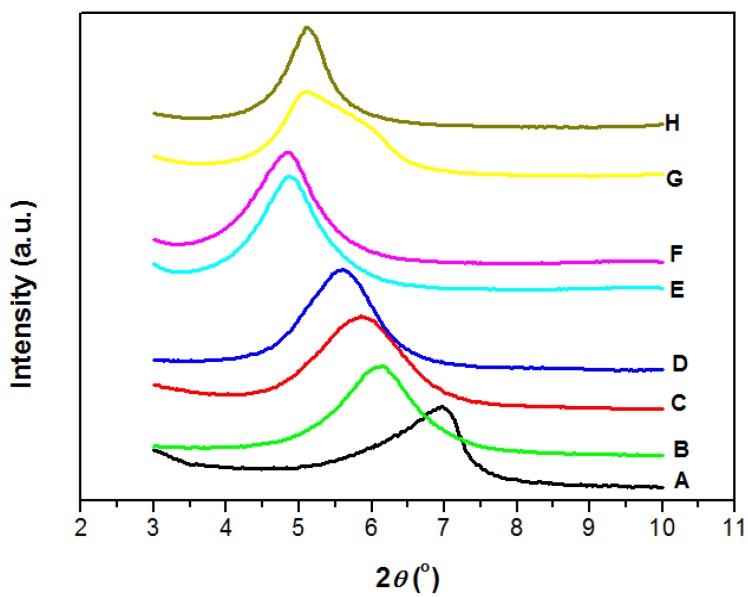
XRD patterns of MMT/PEO hybrids and Pd@MMT/PEO catalytic composites. A. MMT; B. MMT/PEO (90/10); C. MMT/PEO (80/20); D. MMT/PEO (70/30); E. MMT/PEO (60/40); F. MMT/PEO (50/50); G. Pd^2+^@MMT/PEO (0.2/80/20); H. Pd^0^@ MMT/PEO (0.2/80/20).

Figure 1 shows the XRD patterns of MMT/PEO and Pd@MMT/PEO and the basal spacing (*d*_001_) value for the MMT estimated by Bragg’s formula is summarized in Table 1. Meanwhile, when the thickness of the single layer of pure Na^+^-MMT (0.96 nm) is taken into account, the interlayer spacing of MMT can be estimated (also shown in Table 1). In the galleries between the MMT layers, the intercalated PEO chains would show different arrangements according to the interlayer spacing values and macromolecular configurations of PEO chains. For pure Na^+^-MMT, the *d*_001_ is 1.25 nm, and its corresponding interlayer spacing is 0.29 nm. At the MMT/PEO ratios of 90/10, the *d*_001_ and interlayer spacing increases to 1.44 nm and 0.48 nm, respectively. Some of previous works [23,30] showed that the intercalated PEO chains would be in helical conformation. However, it is worth noting that the size of PEO chains with a helical conformation is about 0.8 nm (>0.48 nm). It indicates that the helical conformation of PEO chains in the interlayer space of MMT is not to be the case. Similar with recent work by Zhu et al. [31], lateral single layer of PEO chain with random conformation confined inside the galleries between the MMT layers in the case of MMT/PEO (90/10) might be much more possible. At the MMT/PEO ratios of 80/20 and 70/30, the interlayer spacing shows a slow increase to 0.56 nm (MMT/PEO (80/20)) and 0.62 nm (MMT/PEO (70/30)), respectively. It should be a wider monolayer of PEG chain confined inside the galleries between the MMT layers. At the MMT/PEO ratios further increase to 60/40 and 50/50, the interlayer spacing shows further increases to 0.85 nm (MMT/PEO 60/40) and 0.86 nm (MMT/PEO 50/50), indicating bilayers of PEO chains confined inside the galleries between the MMT layers. As we know, PEO is a polymer with high crystalline capability. One more layers highly-ordered arrangements should be beneficial to the formation of PEO crystals. The formation of crystal phase of PEO bulk would lead to a decrease in organic modification efficiency for MMT. Therefore, MMT/PEO (80/20) with wider monolayer arrangement was chosen as the platform for immobilization of Pd species in preparation of novel heterogeneous catalytic composites. The interlayer spacing of Pd^2+^@MMT/PEO (0.2/80/20) increases to 0.76 nm, 0.2 nm bigger than MMT/PEO (80/20). This confirms that the Pd^2+^ cations are effectively chelated by the PEO chains and well confined inside the galleries of MMT. Though the calculated mean interlayer spacing of Pd^0^@MMT/PEO (0.2/80/20) is close to Pd^2+^@MMT/PEO (0.2/80/20), the scattering peak from Pd^0^@MMT/PEO becomes a bit narrower. It is presumably caused by the local distortion of the layered structure by in-situ-generated Pd^0^ nanoparticles with uniform size.

**Figure 2:**
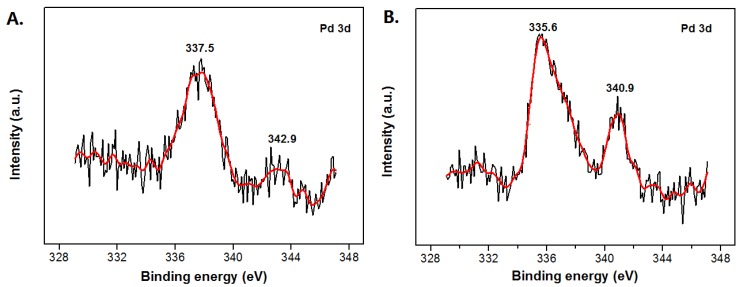
XPS spectra of the Pd^2+^@MMT/PEO (**A**) and Pd^0^@MMT/PEO (**B**) catalytic composites.

**Figure 3:**
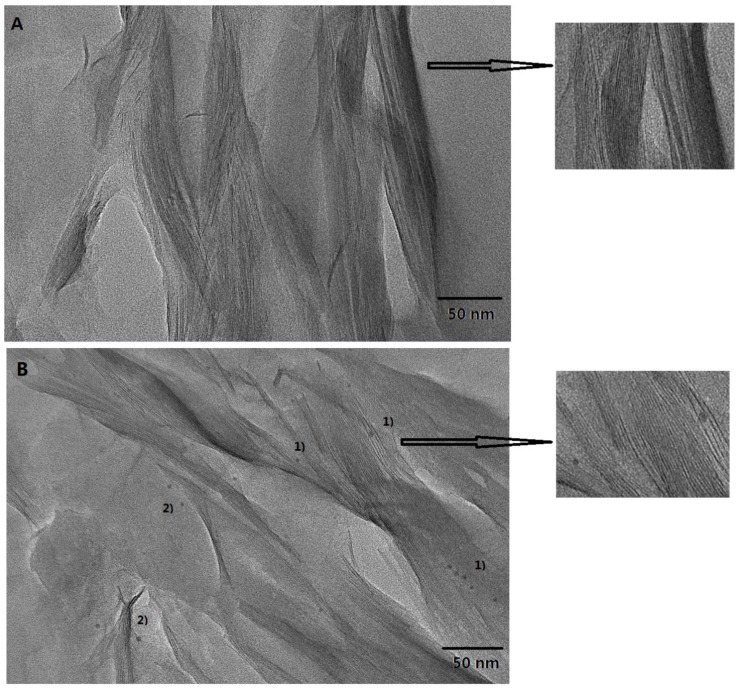
HR-TEM results of the MMT/PEO (80/20) hybrid (**A**) and Pd^0^@MMT/PEO (0.2/80/20) catalytic composite (**B**). The insert figures are the enlarged view of the local regions in the HR-TEM photos.

The microstructure of the Pd@MMT/PEO catalytic composite was further characterized with XPS and HR-TEM. As shown in Figure 2A, for Pd^2+^@MMT/PEO, the binding energy peaks are found at 337.5 eV (Pd3d_5/2_) and 342.9 eV (Pd3d_3/2_), confirming the presences of Pd^2+^ species [32]. As shown in Figure 2B, for Pd^0^@MMT/PEO, the binding energy peaks shift to 335.6 eV (Pd3d_5/2_) and 340.9 eV (Pd3d_3/2_), respectively, confirming the presences of Pd^0^ species [32]. Clearly, XPS characterization results supply a powerful evidence for the conversion of Pd^2+^ to Pd^0^. Figure 3 shows HR-TEM results of the MMT/PEO (80/20) hybrid and Pd^0^@MMT/PEO (0.2/80/20) catalytic composite. MMT/PEO (80/20) shows a typical layered structure with well-ordered parallel stacking of silicate MMT layers. For Pd^0^@MMT/PEO (0.2/80/20), it is observed that the interlayer contrast is much clearer than MMT/PEO (80/20). It should be due to the slight increase in the interlayer spacing after immobilization of Pd species, which is also evidenced by XRD characterization. Meanwhile, it is observed the dispersion states of Pd^0^ nanoparticles (2–4 nm) in the MMT/PEO (80/20) matrices are in two forms: (1) being confined inside 1–3 layers of MMT, (2) dispersed on the outer surface of MMT layers.

Meanwhile, the effects of the Pd immobilization on the molecular level microstructure of Pd@MMT/PEO catalytic composite have been characterized by PALS. The positron annihilation lifetime spectra of MMT/PEO (80/20), Pd^2+^@MMT/PEO (0.2/80/20), and Pd^0^@MMT/PEO (0.2/80/20) was analyzed by three-component fitting using LT-9 and MELT-4 programs. We mainly take attentions to the longest (third) lifetime component (*τ*_3_), which is attributed to the ortho-positronium (*o*-Ps) annihilations inside the interlayer space of MMT [33,34,35]. The size of the micro defects can be estimated from *τ*_3_ with suitable models. It was evidenced that the modified Tao–Eldrup equation for cuboidal voids (Equation (1)) worked well for the MMT-based materials with layered structure [35,36,37,38]. Where, *l* refers to the mean width of cuboidal micro defects, and Δ*l* (0.17 nm) refers to the value of empirical parameter.

(1) $$\tau_{3}=0.5{\left[1-\left(\frac{l}{l+2\Delta l}+\frac{1}{\pi }\sin\frac{\pi l}{l+2\Delta l}\right)\right]}^{-1}$$

**Figure 4:**
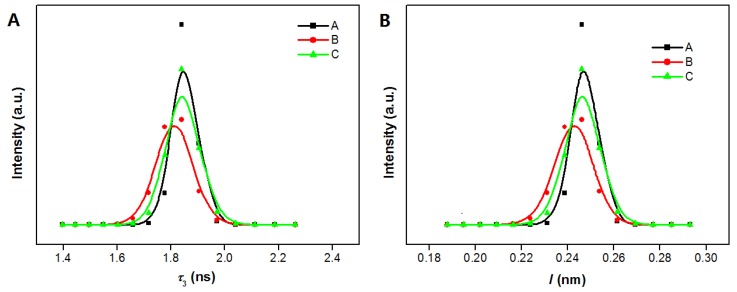
Distribution of the longest lifetime (*τ*_3_) (**A**) and corresponding micro-defect’s size (*l*) (**B**) of the samples analyzed by MELT-4 program. A. MMT/PEO (80/20); B. Pd^2+^@MMT/PEO (0.2/80/20); C. Pd^0^@MMT/PEO (0.2/80/20).

The sizes of the micro defects estimated with Equation (1) are listed in Table 2. The PALS result of pure MMT sample has been reported in our recent work [20], the *o*-Ps lifetime is 2.801 ns, and the mean micro defects size (*l*) of MMT can be calculated as 0.3443 nm. This value is bigger than that of MMT/PEO (80/20), 0.2901 nm. This is due to the fact that the interlayer space of MMT becomes more crowded after the PEO chains intercalate. Meanwhile, the effects of the Pd immobilization and reducing treatment on the microstructure have been sensitively detected. Though the interlayer space is expanded obviously after Pd^2+^ immobilized as determined by XRD, the mean micro defects size (*l*) show a decrease from 0.2901 nm (MMT/PEO (80/20)) to 0.2792 nm (Pd^2+^@MMT/PEO (0.2/80/20)). By forming effective chelation with –OH groups, Pd^2+^ cations play a role similar to cross-linking points for PEO chains, leading to a decrease in micro defects size within the intercalated PEO phase. Pd^0^ species often has poorer chelation capability with polar groups than Pd^2+^ species. Therefore, the micro defects size then undergoes an increase to 0.2886 nm for Pd^0^@MMT/PEO (0.2/80/20). Figure 4 shows the distribution of the longest lifetime (*τ*_3_) and corresponding micro-defect’s size (*l*) of the samples as fitted with MELT-4 program. Similar variation trend of the distribution range of the micro-defect’s size is found.

**Figure 5:**
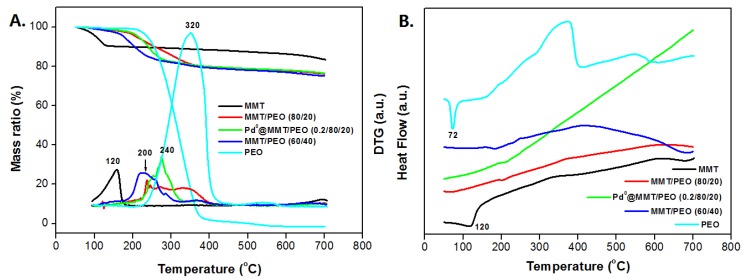
TGA/DTG (**A**) and DSC (**B**) curves of the MMT/PEO hybrids and Pd@MMT/PEO catalytic composite.

Figure 5A shows the TGA/DTG results of MMT/PEO hybrids and Pd^0^@MMT/PEO (0.2/80/20) catalytic composite. As a stable inorganic layered silicate mineral, MMT shows high thermal stability except a weight loss stage caused by the evaporation of absorbed and/or bonded H_2_O before 125 °C. The decomposition temperature (peak temperature of the DTA curve) of PEO is found at about 320 °C. For the MMT/PEO hybrids, decomposition temperature of PEO component shows an obviously decrease to 240 °C for MMT/PEO (80/20) and 200 °C for MMT/PEO (60/40), respectively. This decrease should be due to the different aggregation states of PEO chains between pure PEO and MMT/PEO hybrid. Due to the high crystallization ability, most PEO chains tend to be regularly aggregated to form perfect crystals. As confirmed with DSC curves in Figure 5B, an endothermic peak of PEO crystal at 73 °C is observed. However, as confined in the narrow galleries of MMT, PEO chains have much lower probabilities in regular aggregation to form crystals. Therefore, no obvious endothermic peak has been detected for the MMT/PEO hybrids and Pd^0^@MMT/PEO (0.2/80/20) catalytic composite. Usually, the formation of perfect crystal of PEO chains is advantageous for higher thermal stability. As a result, PEO component in MMT/PEO hybrids shows lower thermal decomposition temperature in TGA curves. The thermal decomposition temperature of Pd^0^@MMT/PEO (0.2/80/20) catalytic composite is close to MMT/PEO (80/20), indicating similar thermal stabilities. Clearly, the prepared Pd^0^@MMT/PEO (0.2/80/20) catalytic composite could be adaptable in organic reactions below 200 °C.

**Figure 6:**
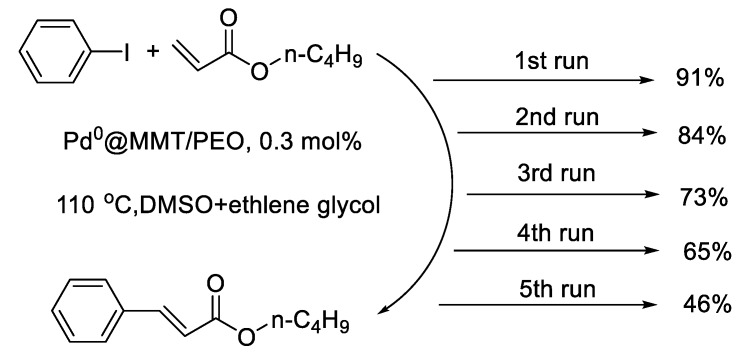
Recycling performances of the Pd^0^@MMT/PEO catalytic composites.

Heck reactions between aryl halides and butyl acrylate were catalyzed with the prepared Pd^0^@MMT/PEO catalytic composite. As shown in Table 3, the Pd^0^@MMT/PEO catalytic composite shows high catalytic activity for the reaction between iodo benzene and *n*-butyl acrylate (entry 1, 91% yield). It still exhibits high catalytic activity for aryl iodides substituted with either an electron-donating group, such as *p*-CH_3_ (entry 2, 89% yield) and *m*-CH_3_O (entry 3, 84% yield), or an electron-absorbing group (such as *p*-F (entry 4, 88% yield), *m*-F (entry 5, 87% yield). Heck reaction between aryl iodides and *t*-butyl acrylate can be also effectively catalyzed with the prepared Pd^0^@MMT/PEO catalytic composite (entry 6–8). The Pd^0^@MMT/PEO catalytic composite show low catalytic activity for the reaction between bromo benzene and *n*-butyl acrylate (entry 9), which is mainly due to the much higher bonding strength of C-Br than C-I to break. Nevertheless, C-Br bond can be activated by substitution of strong electron-absorbing group such as *m*-COCH_3_ (entry 10). Clearly, the catalytic activity of Pd^0^@MMT/PEO is much higher than PEO-supported recyclable NC palladacycle catalysts [28]. And it is also comparable to recent other reported heterogeneous catalysts for Heck reactions [39,40]. After the reaction, the Pd^0^@MMT/PEO catalytic composite can be conveniently separated and recycled for the next run. As shown in Figure 6, the catalytic efficiency of the Pd^0^@MMT/PEO catalytic composite decrease gradually as the recycling times increase. Similarly, higher recyclability is observed for the Pd^0^@MMT/PEO catalytic composite as compared with PEO-supported recyclable NC palladacycle catalysts (can recycle 4 times with moderate yield) [28]. However, it is obviously lower than recent prepared Pd^0^@MMT/PVA or Pd^0^@MMT/PVP catalysts [19,20]. For PEO, the polar –OH groups are distributed in the ending of the chain rather than each repeating unit of the chain like PVA. Reasonably weaker chelation and quicker Pd leaching will occur in the case of Pd^0^@MMT/PEO.

## 4. Conclusions

In this study, PEO chains were successfully intercalated into interlayer spaces of Na^+^-MMT, which can be used as a novel support for Pd^0^ nanoparticles. PEO chains and Pd species are well confined in the interlayer space of MMT, which is well elucidated by XRD, HR-TEM, XPS, TGA, and PALS. It was demonstrated that Pd^0^ nanoparticles sized in 2–4 nm were successfully immobilized on MMT/PEO supports. The sub-nano level micro defects variation of MMT can be sensitively detected by PALS. After PEO intercalation and Pd immobilization, the micro defects undergo a slight decrease in size, which is mainly due to the fact that the interlayer space of MMT becomes more crowded. The prepared Pd^0^@MMT/PEO catalytic composite shows high catalytic activities for Heck reactions and can be recycled for five times. The lower recyclability of the Pd^0^@MMT/PEO than other reported Pd^0^@MMT/polar polymers catalytic composites should be mainly due to the weaker chelation of PEO with Pd. Nevertheless, the comprehensive catalytic performances of the Pd^0^@MMT/PEO are much improved as compared with Pd heterogeneous catalysts which are prepared by directly supporting of Pd species on PEO chains. This work supplies an alternative approach in the preparation of Pd heterogeneous catalysts with fairly good performances, and might have broad prospects in both experimental and industrial applications.

## Figures and Tables

**Table 1:** Results of *d*_001_ spacing and corresponding interlayer spacing (subtracting the thickness of MMT layer of 0.96 nm) of the investigated MMT from XRD.

Sample	2*θ* (°)	*d*_001_ Spacing (nm)	Interlayer Spacing (nm)
A. MMT	7.02	1.25	0.29
B. MMT/PEO (90/10)	6.15	1.44	0.48
C. MMT/PEO (80/20)	5.78	1.52	0.56
D. MMT/PEO (70/30)	5.60	1.58	0.62
E. MMT/PEO (60/40)	4.88	1.81	0.85
F. MMT/PEO (50/50)	4.84	1.82	0.86
G. Pd^2+^@MMT/PEO (0.2/80/20)	5.13	1.72	0.76
H. Pd^0^@MMT/PEO (0.2/80/20)	5.11	1.73	0.77

**Table 2:** Variation of longest lifetime, intensity, and mean size of micro-defects of the samples analyzed by LT-9 program.

Samples	*τ*_3_ (ns)	*I*_3_ (%)	*l* (nm)
MMT/PEO (80/20)	2.235	5.8	0.2901
Pd^2+^@MMT/PEO (0.2/80/20)	2.132	3.7	0.2792
Pd^0^@MMT/PEO (0.2/80/20)	2.220	4.5	0.2886

**Table 3:**
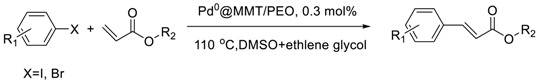
Catalytic performances of Pd^0^@MMT/PEO for Heck reactions between aromatic halides with acrylates.

Entry	Aromatic Halides	Acrylates	Coupling Yield ^a^ (%)
1	C_6_H_5_I	CH_2_=CHCOO(*n*-C_4_H_9_)	91
2	4-CH_3_C_6_H_4_I	CH_2_=CHCOO(*n*-C_4_H_9_)	89
3	3-CH_3_OC_6_H_4_I	CH_2_=CHCOO(*n*-C_4_H_9_)	84
4	4-FC_6_H_4_I	CH_2_=CHCOO(*n*-C_4_H_9_)	88
5	3-FC_6_H_4_I	CH_2_=CHCOO(*n*-C_4_H_9_)	87
6	C_6_H_5_I	CH_2_=CHCOO(*t*-C_4_H_9_)	74
7	4-CH_3_C_6_H_4_I	CH_2_=CHCOO(*t*-C_4_H_9_)	75
8	4-FC_6_H_4_I	CH_2_=CHCOO(*t*-C_4_H_9_)	71
9	C_6_H_5_Br	CH_2_=CHCOO(*n*-C_4_H_9_)	trace ^b^
10	3-COCH_3_C_6_H_4_Br	CH_2_=CHCOO(*n*-C_4_H_9_)	45 ^b^

^a^ GC-MS Yield; ^b^ the reaction time was 10 h.
